# Concentrated and Palatable Powders

**Published:** 1874-02

**Authors:** J. W. Southworth


ART. III.—*Concentrated and Palatable Powders.* A paper read before Toledo Medical Society. By J. W. Southworth, M. D.

*Mr. President and Gentlemen of the Society:* It is with pleasure that I bring to your notice some recently prepared and highly palatable powders of aconite, veratrum, jalap and senna; thus enhancing in some degree the practical utility of our common stock of medicinal agents. These are the only ones yet experimented with, but we are confident that it can be advantageously extended to many others which the wants of the profession may indicate among the arterial or nervous sedatives, narcotics, cathartics, &c.

Our object is to present the virtues of each drug in the form of a powder which shall be the most palatable and compact, as well as stable and soluble condition possible, without materially increasing their cost. By this means the practitioner is enabled to carry an ordinary assortment of drugs in a very small space, and also without the annoyance of breaking vials and the spilling of the liquid contents, nor be troubled to find vials corresponding in size to the broken ones in order to replace them. An ordinary (clasp) cigar case, in which are placed ten small and six large envelopes, (open at the end), made of sheepskin parchment, will afford sufficient space and variety for most physicians who carry much medicine with them to dispense at the bed side of their patients. While to those who carry only a few drugs, the clasp match safe, (a miniature cigar case), will be a most admirable convenience; using, of course, little parchment envelopes—four or five on each side—filled with whatever the exigences of one's practice would call for.

Several physicians of this city have availed themselves of the above plan, Dr. S. S. Thorn being the "pioneer," I believe, in this most excellent method.

It is a well known and deplorable fact that we have not reached that degree of perfection in the art of rendering medicines palatable so much needed, and which is so naturally, earnestly and properly desired by our patients. So much so, that in many cases they turn to the votaries of charlatanism to get the much coveted articles which shall cure (?) them and yet be so very pleasant to take! However inert such remedies *may be*, they of course are illy fitted to judge; and, if for no stronger reason, we should be negligent of our duty to ourselves and to our fellows men, did we not strive to attain the object sought. This; you are all aware, is no equivocal part of our duty as practitioners for it often happens that we are partially, if not wholly, thwarted in our efforts in the treatment of infants and refractory children, or even among fastidious adults by the impalatableness of our usual remedies. I trust none will regard these remarks in the light of an apology for calling your attention to the preparations presented; such an idea would be an injustice to myself as well as to an appreciative, intelligent, and humane profession.

The method of preparation is as follow:—The active principles of the drugs are obtained by either infusion, maceration or percolation with alcohol and water, after which they are concentrated to a proper degree by evaporation and then mixed in definite proportion with sugar of milk and powdered liquorice root, so as to represent when dried and pulverized the entire virtues in as small a bulk and in as palatable a condition as one could wish or expect.

In the case of concentrated aconite powder, 1 gr. is made to equal two minims of the F. E. of Aconite Root. The concentrated verat. viride powder 1 gr. is also made to equal two minims of the F. E. of that plant. Other vegetable, nervous and arterial seda tives, such as gelsemium, digitalis, calabar bean, etc., etc., can be easily prepared in the form of a powder of the same strength as the preceding.

The concentrated jalap powder is condensed to nearly the sam- strength as the powdered extract, and retaining all of its properties *except* that of agglutination. Whilst, if one prefers the jalap resin alone, it can be prepared in a pulverent form, and in as compact a state as could reasonably be desired, the only loss in a therapeutic point of view being the absence of the diuretic action of the drug.

The concentrated powder of senna is prepared by leaving out the liquorice and adding heavy carbonate of magnesia, with or without the same quantity of sugar of milk. My plan is to add the concentrated solution of senna leaves to the sugar of milk until the dried compound was too "gummy" to pulverize properly, when by the addition of the magnesia it could readily be brought to a desirable degree of firmness. The amount of magnesia added was equal in weight to both of the other substances. Lastly, the whole was aromatized with strong tincture of fennel and coriander made from the oils. This makes a most excellent laxative or purgative for children and infants, and is always readily taken in sweetened water. The dose varies from five to ten grains repeated in three or four hours until it operates. By substituting one-half of the magnesia for sulphur we get a much more convenient and compact comp. senna powder than the "Pulvis Glycerrhizæ Compositus" of Dr. David Page (*Practitioner*), which is composed as follows:—℞ Senna leaves Pulv. et Glycerrhizæ Rad. Pulv. aa. ℥vi. + Fennel Sem. Pulv. et Sulph. Flor. aa. ℥iii. + Sacch. Alba pulv. ℥xviii. Mx. Ft. A. powder. Dose, a teaspooful or more at bed time.

If one should wi h, the combina ion of the senna powder with jalap resin would afford a very convenient and reliable laxative or cathartic agent, one that would scarcely be offensive to the taste.

